# Rapid Increase of Oral Bacteria in Nasopharyngeal Microbiota After Antibiotic Treatment in Children With Invasive Pneumococcal Disease

**DOI:** 10.3389/fcimb.2021.744727

**Published:** 2021-10-12

**Authors:** Desiree Henares, Muntsa Rocafort, Pedro Brotons, Mariona F. de Sevilla, Alex Mira, Cristian Launes, Raul Cabrera-Rubio, Carmen Muñoz-Almagro

**Affiliations:** ^1^ Institut de Recerca Sant Joan de Deu, Hospital Sant Joan de Deu, Barcelona, Spain; ^2^ CIBER of Epidemiology and Public Health (CIBERESP), Instituto de Salud Carlos III, Madrid, Spain; ^3^ School of Medicine, Universitat Internacional de Catalunya, Barcelona, Spain; ^4^ Pediatric Department, Hospital Sant Joan de Deu, University of Barcelona, Barcelona, Spain; ^5^ Department of Health and Genomics, Center for Advanced Research in Public Health, Fundacion para el Fomento de la Investigacion Sanitaria y Biomedica de la Comunitat Valenciana (FISABIO), Valencia, Spain; ^6^ Teagasc Food Research Centre (TEAGASC), Moorepark, Fermoy, Ireland; ^7^ APC Microbiome Institute, University College Cork, Cork, Ireland

**Keywords:** children, nasopharyngeal microbiota, invasive pneumococcal disease (IPD), antibiotics, oral bacteria, nosocomial bacteria

## Abstract

**Introduction:**

Antibiotics are commonly prescribed to young children for treating bacterial infections such as invasive pneumococcal disease (IPD) caused by *Streptococcus pneumoniae*. Despite the obvious benefits of antibiotics, little is known about their possible side effects on children’s nasopharyngeal microbiota. In other ecological niches, antibiotics have been described to perturb the balanced microbiota with short- and long-term effects on children’s health. The present study aims to evaluate and compare the nasopharyngeal microbiota of children with IPD and different degree of antibiotic exposure.

**Methods:**

We investigated differences in nasopharyngeal microbiota of two groups of children <18 years with IPD: children not exposed to antibiotics before sample collection (n=27) compared to children previously exposed (n=54). Epidemiological/clinical data were collected from subjects, and microbiota was characterized by Illumina sequencing of V3-V4 amplicons of the 16S rRNA gene.

**Results:**

Main epidemiological/clinical factors were similar across groups. Antibiotic-exposed patients were treated during a median of 4 days (IQR: 3–6) with at least one beta-lactam (100.0%). Higher bacterial richness and diversity were found in the group exposed to antibiotics. Different streptococcal amplicon sequence variants (ASVs) were differentially abundant across groups: antibiotic use was associated to lower relative abundances of *Streptococcus* ASV2 and *Streptococcus* ASV11 (phylogenetically close to *S. pneumoniae*), and higher relative abundances of *Streptococcus* ASV3 and *Streptococcus* ASV12 (phylogenetically close to viridans group streptococci). ASVs assigned to typical bacteria from the oral cavity, including *Veillonella*, *Alloprevotella*, *Porphyromonas*, *Granulicatella*, or *Capnocytophaga*, were associated to the antibiotic-exposed group. Common nosocomial genera such as *Staphylococcus*, *Acinetobacter*, and *Pseudomonas* were also enriched in the group exposed to antibiotics.

**Conclusion:**

Our results point toward a reduction of *S. pneumoniae* abundance on the nasopharynx of children with IPD after antibiotic treatment and a short-term repopulation of this altered niche by oral and nosocomial bacteria. Future research studies will have to evaluate the clinical implications of these findings and if these populations would benefit from the probiotic/prebiotic administration or even from the improvement on oral hygiene practices frequently neglected among hospitalized children.

## Introduction

Antibiotics prescribed for treating infectious diseases save millions of lives every year, but limited information is available about their impact on the human microbiome and consequences on health. The side effects of antibiotics have been described on the gut microbiota, including transient or profound loss of specific bacterial species, reduction of microbial diversity, and loss of colonization resistance (Kim et al., 2017). In hospitalized patients, antibiotic use favors colonization by nosocomial and appearance of multidrug-resistant pathogens thus increasing the risk for healthcare-associated infections (Kim et al., 2017). Even long-term effects on children’s health including obesity or diabetes have been linked to aberrant microbiomes altered by antibiotics (Boursi et al., 2015; Tai et al., 2015).

With respect to the respiratory tract, different authors have reported changes in throat, oropharynx, and lung microbiota of adult patients subjected to long-term antibiotic intake for different infections or chronic respiratory conditions (Hong et al., 2016; Wang et al., 2017; Choo et al., 2018). However, there is scarce research on the effects that occurred on children’s nasopharynx, the ecological niche of the main pathogens causing disease in pediatric populations (Cleary and Clarke, 2017). Moreover, young children are subjected to a high number of short-term antibiotic prescriptions, especially for treating acute respiratory infections (Fleming-Dutra et al., 2016; Orlando et al., 2020) such as invasive pneumococcal disease (IPD).

IPD is a major cause of morbi-mortality worldwide with high incidence among children under 5 years (GBD 2016 Lower Respiratory Infections Collaborators, 2018; Wahl et al., 2018). IPD is caused by *Streptococcus pneumoniae*, a bacteria that normally colonizes the nasopharynx of children asymptomatically but can occasionally cause pneumonia, the most frequent manifestation, or other serious clinical syndromes including sepsis or meningitis (Wahl et al., 2018). Differential susceptibility to IPD might be partly explained by the nasopharyngeal microbiota, which could play a role in the transition of *S. pneumoniae* from colonization to disease states (Camelo-Castillo et al., 2019).

Management of IPD requires antibiotic treatment with varying doses, duration, and administration routes according to clinical and patient’s characteristics (NICE, 2019). Since antibiotics are crucial for appropriate treatment of IPD but also may result in perturbations of the ecological niche of *Streptococcus pneumoniae*, further insight is needed into how this disturbance is produced and what bacteria are leading the repopulation of the nasopharynx so we can counteract antibiotic-derived unwanted effects in our microbiota. Antibiotics side effects may indirectly impact children’s health by causing microbiota imbalances that have been linked to pathogenesis of several respiratory infections (Bosch et al., 2017; Lanaspa et al., 2017) and chronic respiratory disorders (Hahn et al., 2018), or by rising antibiotic-resistant bacteria that have been described to persist for long time and cause infections associated to higher rates of treatment failure and mortality (Sjölund et al., 2005; Jernberg et al., 2007; Jernberg et al., 2010; Friedman et al., 2016). The present study aims to analyze and compare the nasopharyngeal microbiota of children hospitalized with IPD and different degree of exposure to antibiotics.

## Methods

### Study Design, Setting, and Participants

A cross-sectional study was conducted at Sant Joan de Deu Barcelona Children’s Hospital (HSJD) with children prospectively recruited from January 2014 to December 2018. The criteria for inclusion in the study were as follows: 1) <18 years of age; 2) admission to HSJD with clinical suspicion of IPD; 3) microbiological confirmation of IPD by isolation of *S. pneumoniae* and/or DNA detection of *S. pneumoniae* in any normally sterile body fluid (Camelo-Castillo et al., 2019); and 4) nasopharyngeal sample collected at any time during hospital stay for diagnostic or research purposes. Exclusion criteria were not signing informed consent or belonging to a previously defined clinical risk group for developing IPD (Gov.UK, 2020).

Patients not treated or treated only during 24 h before sample collection were considered as cases not exposed to antibiotics, while patients treated for more than 24 h prior to sample collection were considered as cases exposed to antibiotics. Different subjects were included in each group. This criterion was adopted on the basis of previous literature reporting that the sensitivity of molecular-based techniques on respiratory samples is not affected by a relatively low time of exposition to antibiotics (Johansson et al., 2008).

### Sample and Data Collection

Nasopharyngeal aspirates (NPAs) were collected and immediately frozen at -80°C until processed (Camelo-Castillo et al., 2019). Antibiotic types, administration route, and exposure time before and during hospital stay were registered for each case. Relevant epidemiological and other clinical data were recorded from each participant through the parent’s interview or electronic medical record, such as delivery mode or pneumococcal vaccination status (categorized into nonvaccinated children or children ≥1 dose of 7-, 10-, or 13-valent pneumococcal conjugate vaccines (PCVs). Microbiological data were obtained through laboratory analyses. Pneumococcal serotypes 1, 3, 4, 5, 7F, 8, 9A, 9V, 12F, 14, 18C, and 19A were considered as serotypes with high invasive disease potential (Camelo-Castillo et al., 2019). A detailed list and description of the variables collected is included in the metadata file.

### Laboratory Analyses

Bacterial DNA was extracted from NPAs by the automated system NucliSENS easyMag (BioMérieux, Marcy-l’Étoile, France). A duplex real-time PCR targeting *lytA* and *Rnase P* genes was used for pneumococcal DNA detection/quantification (Camelo-Castillo et al., 2019; CDC and Ncird). All positive *S. pneumoniae* samples were further serotyped (Selva et al., 2012). A multiplex Real-Time PCR Anyplex TM II RV16 (Seegene, Seoul, Korea) was used to detect DNA/RNA from 16 human respiratory viruses. Nasopharyngeal microbiota was characterized by 16S rRNA gene sequencing. The V3-V4 region was amplified and sequenced with Illumina MiSeq (Illumina, San Diego, California, USA) as previously described (Illumina 16S Metagenomic Sequencing Library Preparation). To control for potential contaminants, 17 negative controls were extracted, amplified, and sequenced with the samples.

### Bioinformatic and Statistical Analyses

Reads were processed using the DADA2 pipeline (Callahan et al., 2016) obtaining exact amplicon sequence variants (ASVs). ASVs mapping to the human genome (GRCh38) using the Burrow–Wheeler Aligner in Deconseq v0.4.3 were filtered out (Schmieder and Edwards, 2011). Taxonomic annotation of ASVs from kingdom to genus was performed by DADA2 using the Ribosomal Database Project (RDP) training set 16. ASVs were further classified to species by an exact matching approach using function addSpecies from DADA2. Finally, Decontam R package compared prevalence of ASVs in real samples and negative controls (Davis et al., 2018), identifying contaminant ASVs that were removed from downstream analyses.

All statistical analyses were performed with R version 3.6.3. Continuous variables were described as mean and standard deviation (SD) or median and interquartile range (IQR) for parametric and nonparametric variables, respectively. Significance of continuous and normally distributed data was assessed by t-test for group comparisons. In case of nonparametric data, Wilcoxon tests were performed. For categorical data, significance was established through chi-square test or Fisher’s exact test if ≥25% of cells presented expected frequencies ≤5.

Samples were rarefied to minimum sample depth (12,601 sequences) for alpha-diversity analyses. Microbiota richness and diversity were estimated through the calculation of Chao1 and Shannon indices for each rarefied sample using the phyloseq R package (McMurdie and Holmes, 2013), and comparisons by group according to antibiotic exposure were made with linear regression analysis and accounting for confounding factors as copredictors (age, gender, seasonality, vaccination, and severity measured by ICU admission and length of hospital stay). PERMANOVA test from vegan R package (Oksanen et al., 2018) evaluated overall differences on microbiota structure according to antibiotic exposure using a Bray–Curtis matrix of relative abundance data of ASVs. In the PERMANOVA model, we also controlled for the confounding factors described above by including them as covariates. A Random Forest classification model was built in order to identify the most discriminative ASVs between subjects not exposed and exposed to antibiotics using the randomForest R package (Package ‘randomForest’) with default parameters and including all ASVs as explanatory variables as well as confounding variables as covariates. Random Forest is a classification algorithm evolving from the combination of many decision trees. A cross-validation is already built-in in Random Forest, since each tree in the forest has its own training and testing data; each tree uses bootstrapped samples from original data as training set and leaves one-third of data for testing, called out-of-bag (OOB) data. OOB data are used on each tree to predict the outcome, the votes for each predicted outcome from all trees are averaged, and the most voted outcome is selected as the final prediction. Therefore, the out-of-bag error predictions of the classifier were used to calculate the ROC curve and the corresponding area under the curve (AUC) as a measure of the performance of the model. Each predictor variable of the model was given an importance score (mean decrease accuracy), which measures the contribution of each feature to the performance of the model, with higher values indicating higher importance. Specifically, the MDA is the mean decrease in the accuracy over all out-of-bag cross-validated predictions when the values of the variable are randomly permuted after training compared to the original observations. The direction of the association of each quantitative future was estimated *post hoc* with Cliff’s delta test. Finally, most frequent bacterial genera with species implicated in healthcare-associated infections among infants and children were specifically selected in the study (Zingg et al., 2017), and its relative abundances were compared by group using Wilcoxon tests in order to find a possible association with antibiotics.

A phylogenetic tree was constructed containing all streptococcal-type strains from RDP database v11.5 and main streptococcal ASVs detected in our dataset. Cutadapt v1.9 trimmed V3-V4 regions for all the selected RDP streptococcal strains (Martin, 2011), and overall multiple sequence alignment was performed with MAFFT v7.4 (Katoh and Standley, 2013). FastTRee inferred the phylogenetic tree according to maximum-likelihood methods with script make_phylogeny.py (Price et al., 2010).

## Results

### Characteristics of Participants

A total number of 168 cases with IPD were screened for participation in the study. Eighty-one of them met inclusion criteria, with a median age of 32 months (IQR: 18–49) and 58% were male. NPA was collected before receiving antibiotics or within the first 24 h of antibiotic intake in 27 patients (median time=0 days, IQR: 0–0.5), and were assigned to the group not exposed to antibiotics. The 54 remaining inpatients were assigned to the antibiotic-exposed group (median time between the first antibiotic intake and sample collection=4 days, IQR: 3–6). The two groups did not present significant differences either in epidemiological variables or in DNA/RNA viral detections or virulence of pneumococcus causing IPD. Clinical manifestations, days of fever before sample collection, length of hospital stay, and complications were similar among exposed and not-exposed inpatients (**Table 1**).

**Table 1:** Epidemiological, microbiological, and clinical characteristics of the study groups.

	Not exposed (n=27)	Exposed (n=54)	*P*-value^a^
**Epidemiological characteristics**			
Age, months, median (IQR)	33 (19.0-49.5)	28.5 (18.5-48.5)	0.72
Gender, male (%)	12/27 (44.4)	35/54 (64.8)	0.13
Birth weight, grams, mean (sd)^b^	3260 (507)	3358 (444)	0.41
Gestational age, weeks, median (IQR)^c^	40 (38.2-40.4)	40.0 (39.0-40.0)	0.81
House surface per inhabitant, m^2^, median (IQR)^d^	20 (18.1-28.3)	22 (16.7-28.8)	0.87
Seasonality, samples collected during viral season (%)*	17/27 (63.0)	31/54 (57.4)	0.81
Ethnicity, Caucasian (%)	16/24 (66.7)	34/48 (70.8)	0.92
Delivery mode, C-section (%)	6/23 (26.1)	7/42 (16.7)	0.55
Breastfeeding (%)	23/26 (88.5)	41/52 (78.8)	0.36^k^
Breastfeeding duration, months, median (IQR)^e^	6.5 (1.6-12.0)	6.0 (1.0-9.0)	0.41
Schooled (%)	21/26 (80.8)	44/51 (86.3)	0.52^k^
Family members under 5 years (%)	9/25 (36.0)	12/46 (26.1)	0.55
Parental smoking (%)	8/26 (30.8)	18/49 (36.7)	0.79
Basic educational level (%)	3/19 (15.8)	9/43 (20.9)	0.74^k^
≥1 dose of Pneumococcal Conjugate Vaccine (%)	15/27 (55.5)	36/52 (69.2)	0.34
**Microbiological characteristics**			
*** Viral study***			
DNA/RNA viral detection by multiplex PCR (%)	22/27 (81.5)	39/54 (72.2)	0.42^k^
DNA/RNA viral detection >2 viruses by multiplex PCR (%)	9/27 (33.3)	15/54 (27.8)	0.79
Human rhinovirus/enterovirus (%)	16/27 (59.2)	31/53 (58.5)	1.00
Human respiratory syncitial virus (A and B) (%)	4/26 (15.4)	4/53 (7.5)	0.42^k^
Human metapneumovirus (%)	1/27 (3.7)	2/53 (3.8)	1.00^k^
Human coronaviruses (OC43/229E/NL63) (%)	2/27 (7.4)	2/54 (3.7)	0.60^k^
Human parainfluenza viruses (1,2,3,4) (%)	1/27 (3.7)	6/53 (11.3)	0.41^k^
Human influenza viruses (A and B) (%)	3/26 (11.5)	4/53 (7.5)	0.67^k^
Human adenovirus (%)	3/27 (11.1)	8/53 (15.1)	0.74^k^
Human bocavirus (%)	4/25 (16.0)	6/53 (11.3)	0.71^k^
*** Pneumococcal study in invasive samples ^#^***			
Pneumococcal serotype with high invasive disease potential (%)	14/27 (51.8)	18/36 (50.0)	1.00
Pneumococcal serotype covered by PCV13 vaccination (%)	14/27 (51.8)	18/36 (50.0)	1.00
**Clinical characteristics**			
Time of fever before NPA collection, hours, median (IQR)^f^	120 (78-144)	128 (33-192)	0.82
*** Blood analytical parameters at admission***			
C -Reactive Protein, mg/L, median (IQR)^g^	300 (205-324)	268 (146-335)	0.83
Procalcitonin, ng/ml, median (IQR)^h^	11.3 (6.7-17.2)	4.3 (1.2 -15.1)	0.37
Hemoglobin, g/dl, median (IQR)^i^	10.7 (10.1-11.7)	10.8 (9.9-11.6)	0.97
Leukocytes, thousand/mm^3^, mean (SD)^j^	16.8 (8.3)	18.0 (8.9)	0.57
*** Clinical syndromes***			
Complicated pneumonia (%)	16/27 (59.2)	29/54 (53.7)	0.81
Non-complicated pneumonia (%)	7/27 (25.9)	8/54 (14.8)	0.36
Meningitis (%)	3/27 (11.1)	5/54 (9.2)	1.00^k^
Sepsis (%)	0/27 (0.0)	4/54 (7.4)	0.29^k^
Bacteremia (%)	1/27 (3.7)	4/54 (7.4)	0.66^k^
Arthritis (%)	0/27 (0.0)	4/54 (7.4)	0.29^k^
** Hospital stay and complications**			
Length of Hospitalization stay, days, median (IQR)	11.0 (8.0-15.0)	10.0 (6.0-15.0)	0.69
ICU admission (%)	4/27 (14.8)	13/54 (24.1)	0.40^k^
Respiratory support (noninvasive ventilation and/or mechanical ventilation) (%)	2/27 (7.4)	11/54 (20.4)	0.20^k^
Thoracocentesis (%)	10/27 (37.0)	22/54 (40.7)	0.94

a T-test and Wilcoxon test were used for parametric and nonparametric continuous variables, respectively. Chi-square test was used for categorical variables.

b Comparisons performed on 25 not exposed cases and 53 exposed cases.

c Comparisons performed on 26 not exposed cases and 53 exposed cases.

d Comparisons performed on 23 not exposed and 47 exposed cases.

e Comparisons performed on 26 not exposed cases and 50 exposed cases.

f Comparisons performed on 26 not exposed cases and 52 exposed cases.

g Comparisons performed on 26 not exposed cases and 53 exposed cases.

h Comparisons performed on 12 not exposed cases and 25 exposed cases.

i Comparisons performed on 25 not exposed cases and 51 exposed cases.

j Comparisons performed on 25 not exposed cases and 52 exposed cases.

k Fisher exact tests were performed for categorical variables instead of chi-square tests in case of ≥25% of cells presented expected frequencies <5.

*Viral season was defined as the period of time corresponding to Influenza A and VRS circulation over the basal levels according to the Surveillance Plan of ARIs in Catalonia (PIDIRAC) (https://canalsalut.gencat.cat/ca/professionals/vigilancia-epidemiologica/pla-dinformacio-de-les-infeccions-respiratories-agudes-a-catalunya-pidirac/) and reports from the Hospital Surveillance Network for VRS in Catalonia (Vall d’Hebrón Hospital) (https://hospital.vallhebron.com/ca/actualitat/publicacions/informe-xarxa-de-vigilancia-hospitalaria-de-vrs).

^#^A total of three pneumococci could not be serotyped due to low bacterial load.

SD, Standard Deviation; IQR, Interquartile Range; NPA, Nasopharyngeal aspirate.

All subjects exposed to antibiotics before sample collection were treated with beta-lactam antibiotics (100.0%, n=54), and 13 received combined therapy with another antibiotic type (24.1%). Most patients were intravenously administered with antibiotics (n=50, 92.6%), except for four subjects who were exclusively orally treated prior to NPA sample collection.

### Increased Richness and Diversity in the Nasopharynx of Children With IPD Exposed to Antibiotics

A total number of 4,150,123 good quality sequences were obtained from samples and negative controls. This represented a median of 51,309 sequences per sample (IQR: 38,194–73,119) and 468 sequences per negative control (IQR: 192–765) (P<0.001). After contaminant removal, 47,323 sequences per sample (IQR: 32,410–64,945) were kept, corresponding to 3,381 ASVs. A median number of 80 ASVs were detected per sample (IQR: 42–129). **Tables S1**, **S2** show the main ASVs with mean relative abundances over 0.1% in each group.

**Figure 1:**
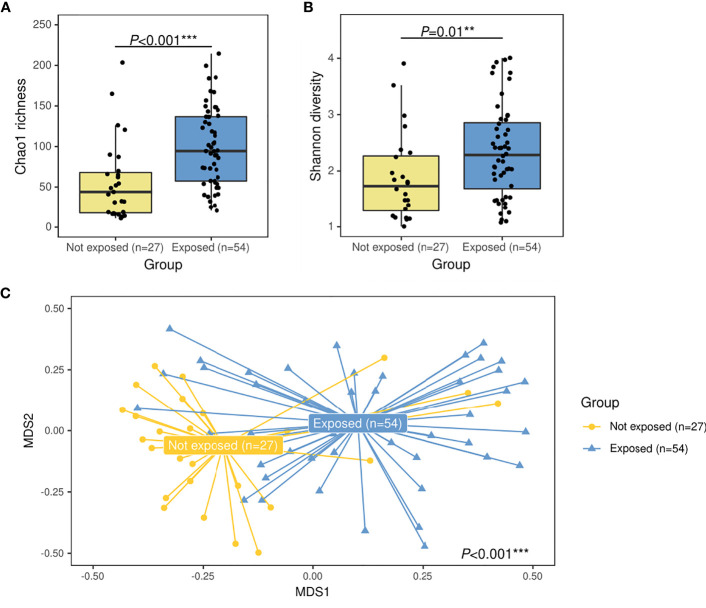
Alpha- and beta-diversity comparisons between patients with IPD not exposed to antibiotics before sample collection (yellow) and IPD patients with previous antibiotic exposure (blue). Boxplots showing the Chao1 richness **(A)** and Shannon diversity indexes **(B)** according to antibiotic-exposure groups at the ASV level. Differences by group were assessed with simple linear regression analyses including confounding variables as copredictors (age, gender, seasonality, vaccination, ICU admission, and length of hospital stay). Three observations from the exposed group were deleted due to missing values in vaccination variable. **(C)** Nonmetric multidimensional scaling (NMDS) plot based on Bray–Curtis dissimilarities of nasopharyngeal microbiota composition of samples from all patients included in the study. Samples of each group are connected with their corresponding centroids using the function “ordispider” (Vegan R package). P-value corresponds to Adonis PERMANOVA test on the antibiotic- exposure group variable and including confounding variables as covariates. Significance codes: *** ≤0.001; ** ≤0.01; * ≤0.05. MDS, nonmetric multidimensional scaling.

For alpha diversity, higher bacterial Chao1 richness (94.8 [IQR: 57.5–137.5] vs. 44.0 [IQR: 17.7–68.0]) (*P*<0.001, R^2^ = 0.20) and higher Shannon diversity (2.2 [IQR: 1.5–2.8] vs. 1.6 [IQR: 1.2–2.1]) (*P*=0.01, R^2^ = 0.18) values were associated to the group exposed to antibiotics (**Figures 1A, B**). For the overall microbiota structure, PERMANOVA analyses further confirmed that both groups presented significant differences on their bacterial composition (P<0.001, R^2^ = 0.03) **Figure 1C**).

### Decreased Abundances of *S. pneumoniae* and Enrichment of Other Streptococci in the Nasopharynx of Children With IPD Exposed to Antibiotics

A specific pneumococcal qPCR demonstrated lower colonization rates (66.7% vs. 100%, *P*=0.002) and lower pneumococcal loads (4.6 [IQR: 0–5.8] log_10_copies/ml vs. 6.3 [IQR: 5.3–6.7] log_10_copies/ml, *P*<0.001) in the nasopharynx of patients exposed to antibiotics vs. not exposed, without significant differences in the invasive disease potential of nasopharyngeal serotypes detected (invasive serotypes; 50.0% *vs.* 51.8%, *P*=1.0) or in their coverage by PCV13 vaccine (50.0% *vs.* 51.8%, *P*=1.0)

**Figure 2:**
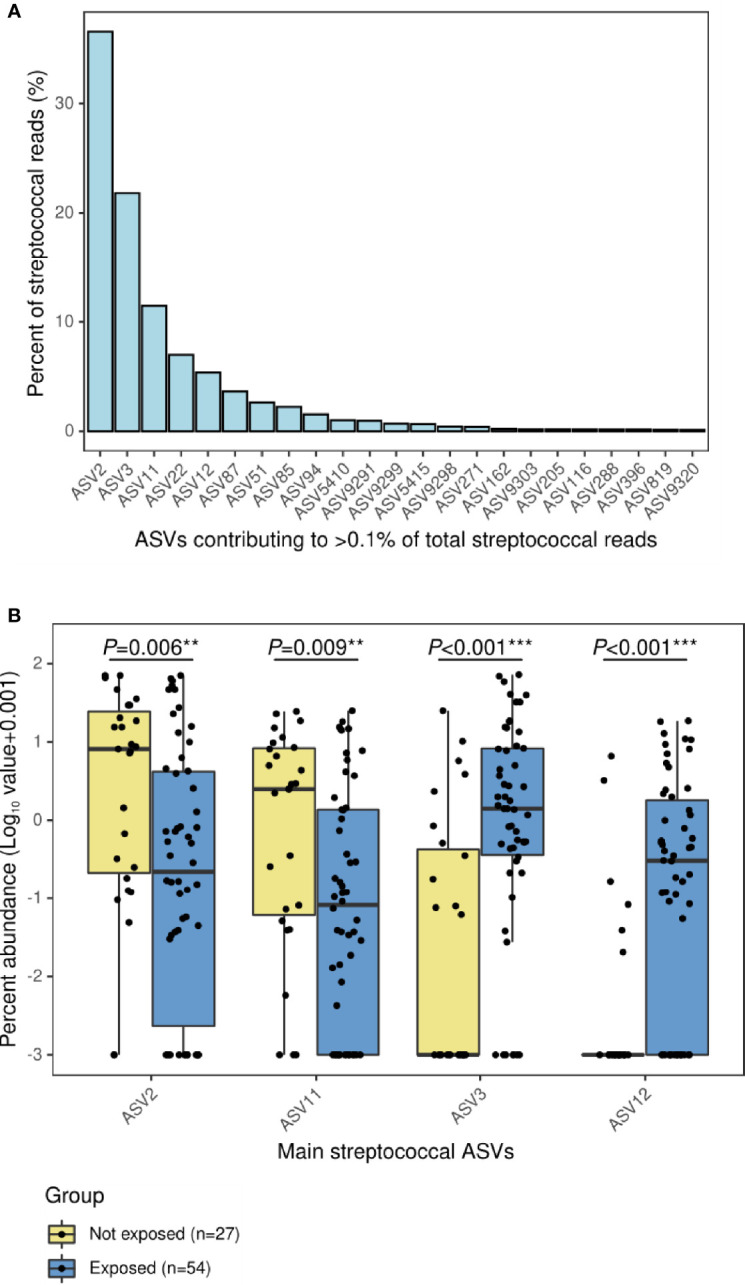
Main ASVs assigned to *Streptococcus* in the nasopharynx of children with IPD and their relative abundance according to antibiotic-exposure groups. **(A)** Bar plot showing the ASVs with a relative contribution >0.1% to the total number of streptococcal reads. **(B)** Boxplot showing the relative abundance of main streptococcal ASVs differentially represented in antibiotic-exposure groups. Significance codes: *** ≤0.001; ** ≤0.01; * ≤0.05.

Analyses with 16S rRNA data not only showed lower abundance of *S. pneumoniae* associated to antibiotic use but also revealed the increase in other streptococci. Overall, a total of 373 ASVs were assigned to *Streptococcus* at the genus level representing the 31.2% of total reads in this dataset. However, 5 ASVs were the most abundant contributing to 25.6% of total reads and 82.2% of the total abundance of reads assigned to *Streptococcus* (**Figure 2A**). When the relative abundances of these five ASVs were compared by group, four of them were differently distributed: the group not exposed to antibiotics was enriched in *Streptococcus* ASV2 (*P*=0.006) and *Streptococcus* ASV11 (*P*=0.009), while exposed patients were enriched in *Streptococcus* ASV3 (*P*<0.001) and *Streptococcus* ASV12 (*P*<0.001) (**Figure 2B**). Interestingly, a streptococcal phylogenetic tree confirmed that *S. pneumoniae* type strain was most closely related to ASV2 and ASV11, while ASV3 and ASV12 were more phylogenetically close to *Streptococcus mitis/oralis/infantis* type strains (**Figure S1**).

### Association of Antibiotic Exposure With Increased Abundance of Oral Bacteria in the Nasopharynx of Children With IPD

**Figure 3:**
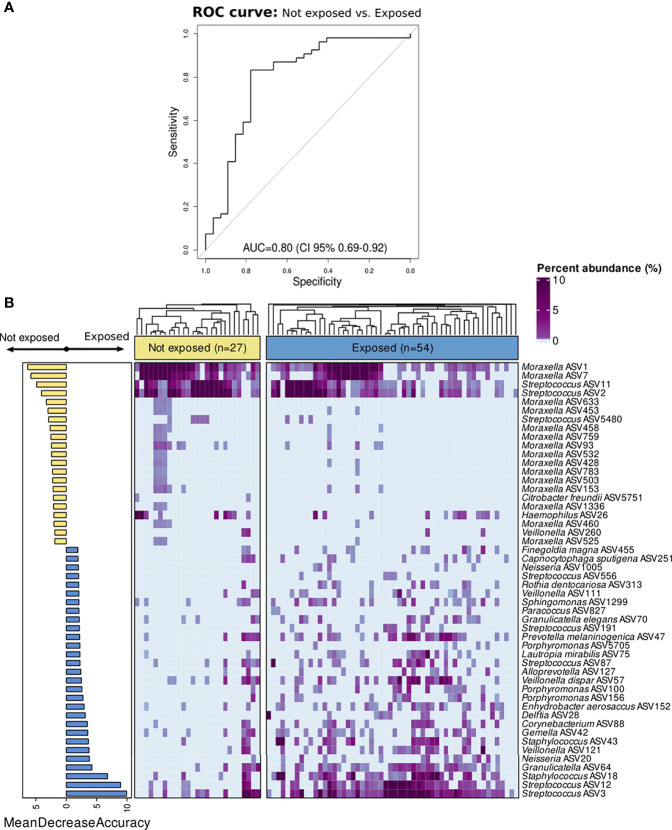
Classification of children with IPD according to antibiotic-exposure using a Random Forest model based on nasopharyngeal microbiota composition. All ASVs as well as confounding factors (age, gender, seasonality, vaccination, ICU admission, and length of hospital stay) were included in the model. **(A)** ROC curve showing the performance of the RF model at the ASV level. **(B)** Bar plot showing top 50 most important features to class separation according to the Mean Decrease Accuracy score (confounding factors were not found among the top important features). Color-coding shows directionality of the association for each of the 50 top features to either the not exposed (yellow) or antibiotic-exposed (blue) group based on a *post hoc* analyses with Cliff’s delta estimation of the effect size. In addition, a heatmap displaying relative abundance (%) of these top 50 features across samples is shown on the right. Each column represents a sample, while each row represents a different feature. In the x-axis, samples are split by group and ordered according to hierarchical clustering using a Bray–Curtis dissimilarity measure.

The results from the Random Forest model demonstrated that nasopharyngeal microbiota composition was different between both groups. The microbiota features exhibited a good discriminatory power for distinguishing inpatients not exposed to antibiotics from those exposed (AUC=0.80 (95% CI:0.69–0.92) (**Figure 3A**). The top 50 features with higher importance in classifying these patients, as measured by the MDA score, were plotted in **Figure 3B**. *Moraxella* ASV1, *Moraxella* ASV7, *Streptococcus* ASV11, and *Streptococcus* ASV2 were found among the top most important features associated to the patients not exposed to antibiotics as determined *post hoc* with Cliff’s delta test. On the contrary, *Streptococcus* ASV3, *Streptococcus* ASV12, *Staphylococcus* ASV18, as well as several ASVs assigned to *Veillonella*, *Alloprevotella*, *Porphyromonas*, *Delftia*, *Capnocytophaga*, *Granulicatella*, *Neisseria*, and other genera were among the top most important features associated to antibiotic use. Most of these genera include gram-negative and anaerobic bacteria frequently found in the oral cavity (Aas et al., 2005; Mira et al., 2017; Dzidic et al., 2018). Further analyses at the genus level located *Prevotella*, *Capnocytophaga*, and *Veillonella* as the most important predictors associated to antibiotic exposure among children with IPD (**Figure S2**). Moreover, differential ranking analysis with Songbird also supported these findings (**Figure S3**).

### Overrepresentation of Common Nosocomial Bacteria Among Children With IPD Exposed to Antibiotics

**Figure 4:**
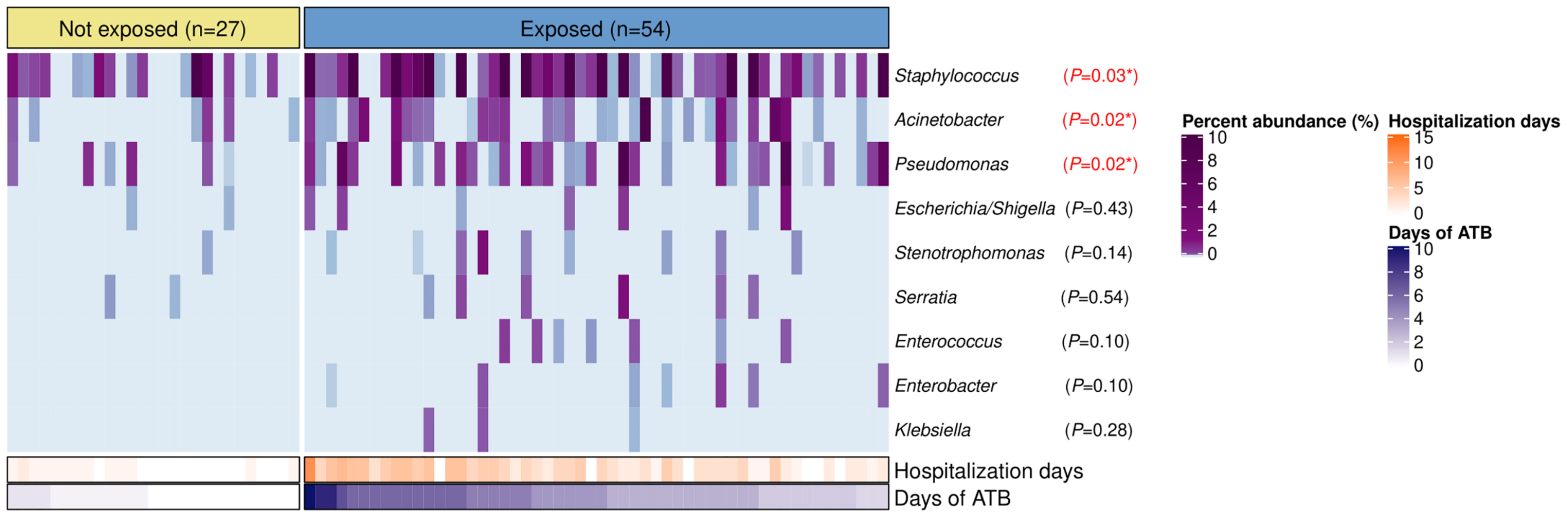
Bacterial genera associated to nosocomial infections in the nasopharynx of patients with IPD according to antibiotic-exposure and length of hospitalization prior to sample collection. Heatmap displaying relative abundances (%) of bacterial genera with characteristic species implicated in healthcare-associated infections. Each column represents a sample, while each row a bacterial genus. Samples are split by the antibiotic-exposure group and ordered by days of antibiotic intake. Rows are sorted by total relative abundance in decreasing order. Bottom bars indicate the number of days of antibiotic exposure and days of hospitalization prior to sample collection for each patient with distinct color scales. Differences in relative abundances of these bacteria by group were assessed with Wilcoxon tests and FDR adjusted p-values are shown. Significance codes: *** ≤0.001; ** ≤0.01; * ≤0.05.

*Staphylococcus*, *Acinetobacter*, *Pseudomonas*, *Escherichia/Shigella*, *Stenotrophomonas*, *Serratia*, *Enterococcus*, *Enterobacter*, *and Klebsiella* were found in the nasopharynx of our patients with total relative abundances of 6.2%, 0.6%, 0.5%, 0.04%, 0.03%, 0.03%, 0.01%, 0.009%, and 0.003%, respectively. However, only *Staphylococcus* (*P*=0.03), *Acinetobacter* (*P*=0.02), and *Pseudomonas* (*P*=0.02) showed significant differences with higher relative abundances in the antibiotic-exposed group (**Figure 4**). Of note, the antibiotic-exposed children also presented longer hospital stays prior to sample collection (median=3 days [IQR: 2–5) vs. median=0 days [IQR=0–1], *P*<0.001).

## Discussion

In the present study, we have demonstrated clear differences in nasopharyngeal microbiota composition of hospitalized children with IPD exposed to antibiotics compared to those not exposed.

Patients exposed to antibiotics presented richer and more diverse microbial nasopharyngeal communities in our study compared to those not exposed. This finding is in contrast to previous literature describing bacterial diversity reduction in gut microbiota as a consequence of antibiotic intake (Kim et al., 2017) and may reflect differential effects of antibiotics across respiratory and intestinal microbiota ecosystems. Our results are in agreement with those reported in a longitudinal study by Smith et al. (2014) in adults with cystic fibrosis. This study analyzed the effects of intravenous beta-lactam antibiotics for treating exacerbations on sputum samples collected on the first day, at 3–4 days, and 8–10 days since antibiotic initiation. A transient increase in diversity at 3 days was described that normalized at days 8–10, suggesting that timing of sampling after the start of antibiotic could be key to understanding the dysbiotic effect of antibiotic exposure in the respiratory microbiota. Results from other investigations on the impact of antibiotics on respiratory microbiota are scarce and heterogeneous, mostly referring to effects observed after at least 7 days of antibiotic initiation: some studies reported decreases in bacterial community complexity (Lazarevic et al., 2013; Pittman et al., 2017; Kramná et al., 2018), while others showed no significant changes on alpha-diversity measures at all (Zhou et al., 2016; Salter et al., 2017). Other factors such as patient age, the specific respiratory condition analyzed, and the respiratory tract segment analyzed may explain the heterogeneity of antibiotic impacts on respiratory microbiota reported so far.

Antibiotic exposure was associated to higher relative abundance of ASVs phylogenetically close to viridans group streptococci (VGS), specifically *Streptococcus mitis/oralis/infantis* species, and anaerobic bacteria such as *Prevotella*, *Aloprevotella*, *Veillonella*, *Porphyromonas*, and *Granulicatella.* These taxa mainly corresponded to commensal bacteria more frequently found in the oropharynx (Aas et al., 2005; Dzidic et al., 2010; Mira et al., 2017) than the nasopharynx of children (Ho Man et al., 2017; SM et al., 2018). Smith et al. (2014) also described a trend for increased relative abundances of anaerobes in sputum samples, mainly *Veillonella* and *Prevotella*, after 72 h of beta-lactam treatment. Similar effects have been observed with pneumococcal vaccination, which resulted in temporary shifts in nasopharyngeal microbiota composition with increased levels of bacterial diversity and increased relative abundances of *Prevotella*, *Veillonella*, unclassified *Bacteroidetes*, and *Leptotrichia* (Biesbroek et al., 2014). Other nonpneumococcal streptococci also raised after vaccination, while pneumococcal-vaccine serotypes decreased. Despite different action mechanisms, both antibiotics and vaccination may lead to eliminating pneumococcus from nasopharynx, with probable displacement of the species detected. Our study suggests that colonizing bacteria from oropharynx may be leading the short-term repopulation of nasopharynx after antibiotic exposure. Although mainly speculative, the poor oral hygiene frequently associated to hospitalized children (Blevins, 2013) is linked to high bacterial loads and increased bacterial colonization (Fourrier et al., 1998; Bordasa et al., 2008; Barbosa et al., 2016; Carrol et al., 2020; Chhaliyil et al., 2020), which may favor the migration of bacteria from oral and dental plaque to the empty nasopharyngeal space left by pneumococcus. Other plausible mechanisms may include the possibility that oral bacteria were already present in the nasopharynx of children with IPD in very low abundance, and such populations expanded to fully occupy the niche after antibiotic use or the new acquisition of oral bacteria *via* breathing, which may have the opportunity to colonize this niche due to the reduction of pneumococcus abundance.

The importance of these findings must be unveiled. Despite the fact that these bacteria are generally commensal microorganisms from the oropharynx (Aas et al., 2005), it has been demonstrated to be a causative role in local and disseminated infections. VGS are a common cause of bacteremia and infective endocarditis (Desai et al., 2017). Gram-negative bacteria such as *Prevotella*, *Porphyromonas*, and *Veillonella* and gram-positive cocci such as *Granulicatella* are isolated from a considerable proportion of mixed anaerobic infections in children (Brook, 2002). In addition, some *Prevotella* and *Porphyromonas* species have been described as important pathogens in periodontitis and inextricably linked to systemic chronic disorders as cardiovascular diseases, diabetes, and rheumatoid arthritis, through cross-reactive antibodies and increased levels of systemic inflammation (Bui et al., 2019).

A higher abundance of VGS and typical oral taxa in the nasopharynx of antibiotic-exposed cases despite active treatment could be explained by the considerable rates of resistance to beta-lactam antibiotics described in these bacteria (Nyfors et al., 2003; Desai et al., 2017; Arredondo et al., 2020), or to the biofilm mode of life of some of these species, which may restrict antibiotic penetration (Kouidhi et al., 2015). In addition, VGS constitute a reservoir of antimicrobial resistance genes that have been described to be transferred to more pathogenic organisms like *S. pneumoniae* (Jensen et al., 2015), while anaerobic gram-negative bacteria may protect penicillin-susceptible bacteria through beta-lactamase production, contributing to antibiotic failures (Brook, 2009). Worrisome is also the fact that antibiotic exposure in children with IPD and hospitalized for a median of 3 days was associated to increased abundances of *Staphylococcus*, *Pseudomonas*, and *Acinetobacter*. These genera, specially *Pseudomonas* and *Acinetobacter*, are not common respiratory commensals and are related to typical multidrug-resistant species causing nosocomial infections (Zingg et al., 2017).

Although it is probable that our findings could be transitory and a recovery of the initial stability of the nasopharyngeal microbiota is reached after antibiotic treatment ends, some changes have been described to persist for long time and for some species to become part of the commensal microbiota. Given the implication of oral bacterial species in multiple disorders and its overrepresentation in the nasopharynx of inpatients with IPD after antibiotic treatment, our study suggests that these patients could benefit from the concurrent administration of probiotics/prebiotics alongside antibiotics that may help to prevent dysbiosis and recover a balanced state of the microbiota (Rosier et al., 2018; Lee et al., 2021). The role of the oral hygiene in preventing and recovering from such disturbances must be revealed. This practice has demonstrated to reduce colonization and density of oral pathogenic bacteria in the oropharynx as well as to reduce the risk of nosocomial infections (Bordasa et al., 2008; Amaral et al., 2009; Carrol et al., 2020; Chhaliyil et al., 2020; Vilela et al., 2015), and could be useful if the repopulation of the nasopharynx of children with IPD after antibiotic treatment is produced by the expansion of oral bacteria from the oropharynx.

This study is subject to a number of limitations. First, the small sample size may have reduced the statistical power. Nevertheless, we would like to note the valuable cohort of patients recruited in a low-prevalence area for IPD and the lack of previous studies assessing the ecological impact of antibiotics in the nasopharynx of these children. Second, its cross-sectional design only allowed identification of associations without establishing causality. Third, 16S rRNA gene sequencing studies cannot differentiate between live and death bacteria. Fourth, analyses at the species levels could not be performed because of the poor taxonomic resolution at this rank, especially for streptococcal species, possibly due to the use of short-length amplicons and the absence of respiratory tract-dedicated, thoroughly curated 16S rRNA gene databases as occurred in gut microbiota. However, we performed analyses at the ASV level that allowed the finest possible resolution by discriminating unique sequences at the single-nucleotide level. Finally, although a phylogenetic tree based on 16S rRNA gene sequences was constructed in order to identify which streptococcal strains were the most phylogenetically close to the streptococcal ASVs detected in our study, other genes may be more suitable for reliable classification of streptococcal species.

Despite these limitations, the associations described here are strong enough to encourage future longitudinal studies that confirm our findings and evaluate the relation of changes observed in nasopharyngeal communities after antibiotic use with short-term and long-term clinical outcomes. Our findings may also encourage shotgun metagenomic studies that help to describe functional profiles and resistance patterns of nasopharyngeal communities associated to antibiotic use.

In conclusion, our results suggest a reduction of *S. pneumoniae* abundance on the nasopharynx of children with IPD after antibiotic treatment, and a short-term repopulation of this altered niche by oral and nosocomial bacteria. This emphasizes the need for understanding the clinical implications of these antibiotic-derived perturbations as well as the utility of probiotic/prebiotic administration or even oral hygiene improvement in preventing or recovering from such disturbances.

## Data Availability Statement

Raw sequence files have been deposited in the European Nucleotide Archive (ENA) at EMBL-EBI under accession number PRJEB46580. The metadata file can be found at FigShare repository (https://figshare.com/s/b52ec8e5a72a49782b77). The rest of data can be found in the article as supplementary files.

## Ethics Statement

This research was approved by the Ethics Committee of HSJD (PIC 70-15 and PIC 137-16) and conducted in compliance with the World Medical Association’s Declaration of Helsinki. All parents and/or legal guardians signed informed consent for children participating in the study.

## Author Contributions

Conception and study design: CM-A and RC-R. Funding: DH and CM-A. Case-control recruitment and data collection: DH, MS, and CL. Microbiological characterization: DH and AM. Bioinformatics analyses/statistical analyses: DH. Guidance in the analyses: MR and RC-R. Interpretation of results: DH, PB, AM, RC-R, and CM-A. Coordination: RC-R and CM-A. Writing manuscript: DH. Revising manuscript: DH, MR, PB, MS, AM, CL, RC-R, and CM-A. All authors contributed to the article and approved the submitted version.

## Funding

This work was supported by Fondo Europeo de Desarrollo Regional (FEDER) and the Ministry of Science and Innovation, Instituto de Salud Carlos III (ISCIII) [PI16/00174 (CM-A), FI17/00248 (DH)], and Sant Joan de Deu Foundation [AFR2015 (CM-A)]. DH also received a grant from Sociedad Española de Enfermedades Infecciosas y Microbiología Clínica (SEIMC) for a research stay. The funders had no role in study design, data collection and interpretation, or the decision to submit the work for publication.

## Conflict of Interest

CM-A reports grants to her organization from Pfizer outside the submitted work and personal fees from Qiagen for presentations at satellite symposiums outside the submitted work.

The remaining authors declare that the research was conducted in the absence of any commercial or financial relationships that could be construed as a potential conflict of interest.

## Publisher’s Note

All claims expressed in this article are solely those of the authors and do not necessarily represent those of their affiliated organizations, or those of the publisher, the editors and the reviewers. Any product that may be evaluated in this article, or claim that may be made by its manufacturer, is not guaranteed or endorsed by the publisher.

## References

[B2] Aas J. A. Paster B. J. Stokes L. N. Olsen I. Dewhirst F. E. (2005). Defining the Normal Bacterial Flora of the Oral Cavity. J. Clin. Microbiol. 43, 5721–5732. doi: 10.1128/JCM.43.11.5721-5732.2005 16272510 PMC1287824

[B3] Arredondo A. Blanc V. Mor C. Nart J. León R. (2020). Resistance to β-Lactams and Distribution of β-Lactam Resistance Genes in Subgingival Microbiota From Spanish Patients With Periodontitis. Clin. Oral. Investig. 24, 4639–4648. doi: 10.1007/s00784-020-03333-1 32495224

[B56] Amaral S. M. Cortês A. Q. Pires F. R. (2009). Nosocomial Pneumonia: Importance of the Oral Environment. J. Bras. Pneumol. 35, 1116–1124. doi: 10.1590/S1806-37132009001100010 20011848

[B16] Barbosa C. G. Leme T. D. Barbosa A. G. Miranda A. F. Piemonte J. A. Barreto A. C. (2016). Oral Microbial Colonization in Pediatric Intensive Care Unit Patients. J. Dent. Child. (Chic) 83, 53–59. 27620514

[B4] Biesbroek G. Wang X. Keijser B. J. F. Eijkemans R. M. J. Trzciński K. Rots N. Y. (2014). Seven-Valent Pneumococcal Conjugate Vaccine and Nasopharyngeal Microbiota in Healthy Children. Emerg. Infect. Dis. 20, 201–210. doi: 10.3201/eid2002.131220 24447437 PMC3901477

[B5] Blevins J. Y. (2013). Status of Oral Health Care in Hospitalized Children. MCN Am. J. Matern. Nurs. 38, 115–119. doi: 10.1097/NMC.0b013e318269daac 23426054

[B1] Bordasa A. McNaba R. Staplesb A. M. Bowmanb J. Kanapkab J. Bosmaa M. P. (2008). Impact of Different Tongue Cleaning >Methods on the Bacterial Load of the Tongue Dorsum. Arch. Oral. Biol. 53 (Suppl 1), S13–S18. doi: 10.1016/S0003-9969(08)70004-9 18460399

[B6] Bosch A. A. T. M. De Steenhuijsen Piters W. A. A. Van Houten M. A. Chu M. L. J. N. Biesbroek G. Kool J. (2017). Maturation of the Infant Respiratory Microbiota, Environmental Drivers, and Health Consequences. Am. J. Respir. Crit. Care Med. 196, 1582–1590. doi: 10.1164/rccm.201703-0554OC 28665684

[B7] Boursi B. Mamtani R. Haynes K. Yang Y. X. (2015). The Effect of Past Antibiotic Exposure on Diabetes Risk. Eur. J. Endocrinol. 172, 639–648. doi: 10.1530/EJE-14-1163 25805893 PMC4525475

[B8] Brook I. (2002). Anaerobic Infections in Children. Microbes Infect. 4, 1271–1280. doi: 10.1016/S1286-4579(02)01656-8 12467770

[B9] Brook I. (2009). The Role of Beta-Lactamase-Producing-Bacteria in Mixed Infections. BMC Infect. Dis. 9, 202. doi: 10.1186/1471-2334-9-202 20003454 PMC2804585

[B10] Bui F. Q. Almeida-da-Silva C. L. C. Huynh B. Trinh A. Liu J. Woodward J. (2019). Association Between Periodontal Pathogens and Systemic Disease. Biomed. J. 42, 27–35. doi: 10.1016/j.bj.2018.12.001 30987702 PMC6468093

[B12] Callahan B. J. McMurdie P. J. Rosen M. J. Han A. W. Johnson A. J. A. Holmes S. P. (2016). DADA2: High-Resolution Sample Inference From Illumina Amplicon Data. Nat. Methods 13, 581–583. doi: 10.1038/nmeth.3869 27214047 PMC4927377

[B13] Camelo-Castillo A. Henares D. Brotons P. Galiana A. Rodríguez J. C. Mira A.. (2019). Nasopharyngeal Microbiota in Children With Invasive Pneumococcal Disease: Identification of Bacteria With Potential Disease-Promoting and Protective Effects. Front. Microbiol. 10, 11. doi: 10.3389/fmicb.2019.00011 30745895 PMC6360994

[B14] Carrol D. H. Chassagne F. Dettweiler M. Quave C. L. (2020). Antibacterial Activity of Plant Species Used for Oral Health Against Porphyromonas Gingivalis. PloS One 15, e0239316. doi: 10.1371/JOURNAL.PONE.0239316 33031410 PMC7544490

[B15] CDC Ncird Laboratory Methods for the Diagnosis of Meningitis - CHAPTER 8: Identification and Characterization of Streptococcus Pneumoniae. Available at: http://www.cdc.gov/ncidod/biotech/strep/strep-doc/index.htm (Accessed August 27, 2021).

[B17] Chhaliyil P. Fischer K. Schoel B. Chhalliyil P. (2020). Impact of Different Bedtime Oral Cleaning Methods on Dental-Damaging Microbiota Levels. Dent. Hypotheses 11, 40–46. doi: 10.4103/denthyp.denthyp_7_20

[B18] Choo J. M. Abell G. C. J. Thomson R. Morgan L. Waterer G. Gordon D. L. (2018). Impact of Long-Term Erythromycin Therapy on the Oropharyngeal Microbiome and Resistance Gene Reservoir in Non-Cystic Fibrosis Bronchiectasis. mSphere 3, e00103–e00118. doi: 10.1128/msphere.00103-18 29669883 PMC5907653

[B19] Cleary D. W. Clarke S. C. (2017). The Nasopharyngeal Microbiome. Emerg. Top. Life Sci. 1, 297–312. doi: 10.1042/ETLS20170041 33525776

[B20] Davis N. M. Proctor Di M. Holmes S. P. Relman D. A. Callahan B. J. (2018). Simple Statistical Identification and Removal of Contaminant Sequences in Marker-Gene and Metagenomics Data. Microbiome 6, 226. doi: 10.1186/s40168-018-0605-2 30558668 PMC6298009

[B21] Desai N. Steenbergen J. Katz D. E. (2017). “Antibiotic Resistance of Non-Pneumococcal Streptococci and Its Clinical Impact,” in Antimicrobial Drug Resistance (Switzerland: Springer International Publishing), 791–810. doi: 10.1007/978-3-319-47266-9_2

[B22] Dzidic M. Collado M. C. Abrahamsson T. Artacho A. Stensson M. Jenmalm M. C. (2018). Oral Microbiome Development During Childhood: An Ecological Succession Influenced by Postnatal Factors and Associated With Tooth Decay. ISME J. 12, 2292–2306. doi: 10.1038/s41396-018-0204-z 29899505 PMC6092374

[B24] Fleming-Dutra K. E. Hersh A. L. Shapiro D. J. Bartoces M. Enns E. A. File T. M.. (2016). Prevalence of Inappropriate Antibiotic Prescriptions Among Us Ambulatory Care Visits 2010-2011. JAMA - J. Am. Med. Assoc. 315, 1864–1873. doi: 10.1001/jama.2016.4151 27139059

[B23] Fourrier F. Duvivier B. Boutigny H. Roussel-Delvallez M. Chopin C. (1998). Colonization of Dental Plaque: A Source of Nosocomial Infections in Intensive Care Unit Patients. Crit. Care Med. 26, 301–308. doi: 10.1097/00003246-199802000-00032 9468169

[B44] Friedman N. D. Temkin E. Carmeli Y. (2016). The Negative Impact of Antibiotic Resistance. Clin. Microbiol. Infect. 22, 416–422. doi: 10.1016/J.CMI.2015.12.002 26706614

[B25] GBD 2016 Lower Respiratory Infections Collaborators (2018). Estimates of the Global, Regional, and National Morbidity, Mortality, and Aetiologies of Lower Respiratory Infections in 195 Countries 1990-2016: A Systematic Analysis for the Global Burden of Disease Study 2016. Lancet Infect. Dis. 18, 1191–1210. doi: 10.1016/S1473-3099(18)30310-4 30243584 PMC6202443

[B49] Gov.UK (2020). Pneumococcal: The Green Book, Chapter 25 - GOV.Uk. Available at: https://www.gov.uk/government/publications/pneumococcal-the-green-book-chapter-25 (Accessed August 27, 2021).

[B26] Hahn A. Warnken S. Pérez-Losada M. Freishtat R. J. Crandall K. A. (2018). Microbial Diversity Within the Airway Microbiome in Chronic Pediatric Lung Diseases. Infect. Genet. Evol. 63, 316. doi: 10.1016/J.MEEGID.2017.12.006 29225146 PMC5992000

[B27] Ho Man W. de Steenhuijsen Piters W. A. Bogaert D. (2017). The Microbiota of the Respiratory Tract: Gatekeeper to Respiratory Health. Nat. Rev. Microbiol. 15, 259–270. doi: 10.1038/nrmicro.2017.14 28316330 PMC7097736

[B28] Hong B. Y. Maulén N. P. Adami A. J. Granados H. Balcells M. E. Cervantes J. (2016). Microbiome Changes During Tuberculosis and Antituberculous Therapy. Clin. Microbiol. Rev. 29, 915–926. doi: 10.1128/CMR.00096-15 27608937 PMC5010754

[B29] Illumina 16S Metagenomic Sequencing Library Preparation (15044223 B) Available at: https://support.illumina.com/content/dam/illumina-support/documents/documentation/chemistry_documentation/16s/16s-metagenomic-library-prep-guide-15044223-b.pdf (Accessed August 27, 2021).

[B30] Jensen A. Valdórsson O. Frimodt-Møller N. Hollingshead S. Kilian M. (2015). Commensal Streptococci Serve as a Reservoir for β-Lactam Resistance Genes in Streptococcus Pneumoniae. Antimicrob. Agents Chemother. 59, 3529–3540. doi: 10.1128/AAC.00429-15 25845880 PMC4432199

[B31] Jernberg C. Lö S. Edlund C. Jansson J. K. Se C. J. (2010). Long-Term Impacts of Antibiotic Exposure on the Human Intestinal Microbiota. Microbiology 156, 3216–3223. doi: 10.1099/mic.0.040618-0 20705661

[B11] Jernberg C. Löfmark S. Edlund C. Jansson J. K. (2007). Long-Term Ecological Impacts of Antibiotic Administration on the Human Intestinal Microbiota. ISME J. 1, 56–66. doi: 10.1038/ISMEJ.2007.3 18043614

[B32] Johansson N. Kalin M. Giske C. G. Hedlund J. (2008). Quantitative Detection of Streptococcus Pneumoniae From Sputum Samples With Real-Time Quantitative Polymerase Chain Reaction for Etiologic Diagnosis of Community-Acquired Pneumonia. Diagn. Microbiol. Infect. Dis. 60, 255–261. doi: 10.1016/j.diagmicrobio.2007.10.011 18036762

[B33] Katoh K. Standley D. M. (2013). MAFFT Multiple Sequence Alignment Software Version 7: Improvements in Performance and Usability. Mol. Biol. Evol. 30, 772–780. doi: 10.1093/molbev/mst010 23329690 PMC3603318

[B34] Kim S. Covington A. Pamer E. G. (2017). The Intestinal Microbiota: Antibiotics, Colonization Resistance, and Enteric Pathogens. Immunol. Rev. 279, 90–105. doi: 10.1111/imr.12563 28856737 PMC6026851

[B35] Kouidhi B. Al Qurashi Y. M. A. Chaieb K. (2015). Drug Resistance of Bacterial Dental Biofilm and the Potential Use of Natural Compounds as Alternative for Prevention and Treatment. Microb. Pathog. 80, 39–49. doi: 10.1016/j.micpath.2015.02.007 25708507

[B36] Kramná L. Dřevínek P. Lin J. Kulich M. Cinek O. (2018). Changes in the Lung Bacteriome in Relation to Antipseudomonal Therapy in Children With Cystic Fibrosis. Folia Microbiol. (Praha) 63, 237–248. doi: 10.1007/s12223-017-0562-3 29127619

[B37] Lanaspa M. Bassat Q. Medeiros M. M. Muñoz-Almagro C. (2017). Respiratory Microbiota and Lower Respiratory Tract Disease. Expert Rev. Anti Infect. Ther. 15, 703–711. doi: 10.1080/14787210.2017.1349609 28661199

[B38] Lazarevic V. Manzano S. Gaïa N. Girard M. Whiteson K. Hibbs J. (2013). Effects of Amoxicillin Treatment on the Salivary Microbiota in Children With Acute Otitis Media. Clin. Microbiol. Infect. 19, e335–e342. doi: 10.1111/1469-0691.12213 23565884

[B39] Lee C. H. Choi Y. Seo S. Y. Kim S. H. Kim I. H. Kim S. W. (2021). Addition of Probiotics to Antibiotics Improves the Clinical Course of Pneumonia in Young People Without Comorbidities: A Randomized Controlled Trial. Sci. Rep. 11, 1–9. doi: 10.1038/s41598-020-79630-2 33441702 PMC7806890

[B41] Martin M. (2011). Cutadapt Removes Adapter Sequences From High-Throughput Sequencing Reads. EMBnet.journal 17, 10. doi: 10.14806/ej.17.1.200

[B42] McMurdie P. J. Holmes S. (2013). Phyloseq: An R Package for Reproducible Interactive Analysis and Graphics of Microbiome Census Data. PloS One 8, e61217. doi: 10.1371/journal.pone.0061217 23630581 PMC3632530

[B43] Mira A. Simon-Soro A. Curtis M. A. (2017). Role of Microbial Communities in the Pathogenesis of Periodontal Diseases and Caries. J. Clin. Periodontol. 44, S23–S38. doi: 10.1111/jcpe.12671 28266108

[B50] NICE (2019). Pneumonia (Community-Acquired): Antimicrobial Prescribing NICE Guideline. Available at: www.nice.org.uk/guidance/ng138 (Accessed August 27, 2021).

[B45] Nyfors S. Könönen E. Bryk A. Syrjänen R. Jousimies-Somer H. (2003). Age-Related Frequency of Penicillin Resistance of Oral Veillonella. Diagn. Microbiol. Infect. Dis. 46, 279–283. doi: 10.1016/S0732-8893(03)00082-8 12944020

[B46] Oksanen J. Blanchet F. G. Friendly M. Kindt R. Legendre P. Mcglinn D. (2018) Title Community Ecology Package. Available at: https://cran.r-project.org/web/packages/vegan/vegan.pdf (Accessed August 27, 2021).

[B47] Orlando V. Monetti V. M. Juste A. M. Russo V. Mucherino S. Trama U. (2020). Drug Utilization Pattern of Antibiotics: The Role of Age, Sex and Municipalities in Determining Variation. Risk Manage. Healthc. Policy 13, 63–71. doi: 10.2147/RMHP.S223042 PMC6996207 32099490

[B100] Package ‘randomForest’. (Available at: https://cran.r-project.org/web/packages/randomForest/randomForest.pdf (Accessed August 27, 2021).

[B48] Pittman J. E. Wylie K. M. Akers K. Storch G. A. Hatch J. Quante J. (2017). Association of Antibiotics, Airway Microbiome, and Inflammation in Infants With Cystic Fibrosis. Ann. Am. Thorac. Soc 14, 1548–1555. doi: 10.1513/AnnalsATS.201702-121OC 28708417 PMC5718571

[B51] Price M. N. Dehal P. S. Arkin A. P. (2010). FastTree 2 - Approximately Maximum-Likelihood Trees for Large Alignments. PloS One 5, e9490. doi: 10.1371/journal.pone.0009490 20224823 PMC2835736

[B52] Rosier B. T. Marsh P. D. Mira A. (2018). Resilience of the Oral Microbiota in Health: Mechanisms That Prevent Dysbiosis. J. Dent. Res. 97, 371–380. doi: 10.1177/0022034517742139 29195050

[B53] Salter S. J. Turner C. Watthanaworawit W. de Goffau M. C. Wagner J. Parkhill J. (2017). A Longitudinal Study of the Infant Nasopharyngeal Microbiota: The Effects of Age, Illness and Antibiotic Use in a Cohort of South East Asian Children. PloS Negl. Trop. Dis. 11, 10. doi: 10.1371/journal.pntd.0005975 PMC5638608 28968382

[B54] Schmieder R. Edwards R. (2011). Fast Identification and Removal of Sequence Contamination From Genomic and Metagenomic Datasets. PloS One 6, e17288. doi: 10.1371/journal.pone.0017288 21408061 PMC3052304

[B55] Selva L. del Amo E. Brotons P. Muñoz-Almagro C. (2012). Rapid and Easy Identification of Capsular Serotypes of Streptococcus Pneumoniae by Use of Fragment Analysis by Automated Fluorescence-Based Capillary Electrophoresis. J. Clin. Microbiol. 50, 3451–3457. doi: 10.1128/JCM.01368-12 22875895 PMC3486242

[B40] Sjölund M. Tano E. Blaser M. J. Andersson D. I. Engstrand L. (2005). Persistence of Resistant Staphylococcus Epidermidis After Single Course of Clarithromycin. Emerg. Infect. Dis. 11, 1389–1393. doi: 10.3201/EID1109.050124 16229767 PMC3310621

[B58] Smith D. J. Badrick A. C. Zakrzewski M. Krause L. Bell S. C. Anderson G. J. (2014). Pyrosequencing Reveals Transient Cystic Fibrosis Lung Microbiome Changes With Intravenous Antibiotics. Eur. Respir. J. 44, 922–930. doi: 10.1183/09031936.00203013 25034564

[B59] Tai N. Wong F. S. Wen L. (2015). The Role of Gut Microbiota in the Development of Type 1, Type 2 Diabetes Mellitus and Obesity. Rev. Endocr. Metab. Disord. 16, 55–65. doi: 10.1007/s11154-015-9309-0 25619480 PMC4348024

[B57] Teo S. M. Tang H. H. F. Mok D. Judd L. M. Watts S. C. Pham K. (2018). Airway Microbiota Dynamics Uncover a Critical Window for Interplay of Pathogenic Bacteria and Allergy in Childhood Respiratory Disease. Cell Host Microbe 24, 341–352.e5. doi: 10.1016/J.CHOM.2018.08.005 30212648 PMC6291254

[B60] Vilela M. C. N. Ferreira G. Z. Santos P. S. D. S. Rezende N. P. M. D. (2015). Oral Care and Nosocomial Pneumonia: A Systematic Review. Einstein (São Paulo) 13, 290–296. doi: 10.1590/S1679-45082015RW2980 25946053 PMC4943826

[B61] Wahl B. O’Brien K. L. Greenbaum A. Majumder A. Liu L. Chu Y. (2018). Burden of Streptococcus Pneumoniae and Haemophilus Influenzae Type B Disease in Children in the Era of Conjugate Vaccines: Global, Regional, and National Estimates for 2000–15. Lancet Glob. Heal. 6, e744–e757. doi: 10.1016/S2214-109X(18)30247-X PMC6005122 29903376

[B62] Wang Z. J. Chen X. F. Zhang Z. X. Li Y. C. Deng J. Tu J. (2017). Effects of Anti-Helicobacter Pylori Concomitant Therapy and Probiotic Supplementation on the Throat and Gut Microbiota in Humans. Microb. Pathog. 109, 156–161. doi: 10.1016/j.micpath.2017.05.035 28552806

[B63] Zhou Y. Bacharier L. B. Isaacson-Schmid M. Baty J. Schechtman K. B. Sajol G. (2016). Azithromycin Therapy During Respiratory Syncytial Virus Bronchiolitis: Upper Airway Microbiome Alterations and Subsequent Recurrent Wheeze. J. Allergy Clin. Immunol. 138, 1215–1219.e5. doi: 10.1016/j.jaci.2016.03.054 27339392 PMC5056860

[B64] Zingg W. Hopkins S. Gayet-Ageron A. Holmes A. Sharland M. Suetens C. (2017). Health-Care-Associated Infections in Neonates, Children, and Adolescents: An Analysis of Paediatric Data From the European Centre for Disease Prevention and Control Point-Prevalence Survey. Lancet Infect. Dis. 17, 381–389. doi: 10.1016/S1473-3099(16)30517-5 28089444

