# Supplementary material for: Rapid Increase of Oral Bacteria in Nasopharyngeal Microbiota After Antibiotic Treatment in Children With Invasive Pneumococcal Disease
Source: Front Cell Infect Microbiol. 2021 Oct 12;11:744727. doi: 10.3389/fcimb.2021.744727 (PMC8546175; doi:10.3389/fcimb.2021.744727)

Table S1. List of ASVs detected with mean relative abundances (%) over 0.1% in the group not exposed to antibiotics (n=27).

ASV name	Not exposed (mean Relative Abundance %)	Not exposed (sd)
ASV1_Moraxella	17.40	22.87
ASV2_Streptococcus	17.15	22.54
ASV10_Haemophilus_influenzae	8.53	18.67
ASV7_Moraxella	5.75	7.52
ASV11_Streptococcus	5.51	7.24
ASV5_Moraxella_nonliquefaciens	5.14	14.13
ASV26_Haemophilus	4.27	14.33
ASV4_Haemophilus	2.74	8.60
ASV51_Streptococcus_pneumoniae	2.46	9.73
ASV30_Haemophilus	2.34	4.91
ASV3_Streptococcus	1.82	5.16
ASV13_Moraxella	1.48	4.29
ASV699_Neisseria	1.26	6.56
ASV5408_Haemophilus	1.20	4.14
ASV9_Haemophilus	1.15	5.75
ASV16_Haemophilus_influenzae	0.93	2.76
ASV9291_Streptococcus	0.90	3.69
ASV1146_Phyllobacterium	0.88	4.55
ASV6_Dolosigranulum_pigrum	0.84	2.37
ASV19_Moraxella	0.80	3.89
ASV14_Haemophilus	0.61	1.82
ASV5415_Streptococcus	0.61	3.16
ASV8641_Moraxella_nonliquefaciens	0.56	2.91
ASV8_Moraxella_lincolnii	0.54	2.01
ASV78_Haemophilus_influenzae	0.51	2.27
ASV34_Haemophilus_influenzae	0.44	1.47
ASV393_Neisseria	0.41	2.12
ASV29_Haemophilus	0.39	1.98
ASV22_Streptococcus_pneumoniae	0.39	2.01
ASV31_Haemophilus_influenzae	0.38	1.93
ASV12_Streptococcus	0.37	1.38
ASV102_Neisseria	0.34	1.65
ASV37_Staphylococcus	0.34	1.09
ASV23_Corynebacterium	0.33	0.90
ASV57_Veillonella_dispar	0.32	0.83
ASV5429_Neisseria	0.31	1.61
ASV1117_Haemophilus_haemolyticus	0.30	1.54
ASV258_Haemophilus	0.30	1.54
ASV64_Granulicatella	0.27	0.77
ASV47_Prevotella_melaninogenica	0.26	0.65
ASV17_Dolosigranulum	0.26	0.81
ASV18_Staphylococcus	0.26	1.07
ASV118_Haemophilus_parainfluenzae	0.20	0.96
ASV85_Streptococcus_peroris	0.19	0.93

ASV94_Streptococcus_salivarius	0.19	0.97
ASV271_Streptococcus_sanguinis	0.19	0.93
ASV121_Veillonella	0.17	0.63
ASV8638_Haemophilus	0.16	0.58
ASV606_Caulobacter	0.16	0.82
ASV114_Alloprevotella	0.15	0.76
ASV89_Alloprevotella	0.12	0.43
ASV21_Moraxella	0.12	0.63
ASV260_Veillonella	0.12	0.46
ASV197_Fusobacterium	0.11	0.53
ASV46_Staphylococcus	0.10	0.27
ASV42_Gemella	0.10	0.35
ASV109_Moraxella	0.10	0.53

Abbreviations: ASV; Amplicon Sequence Variant. SD; Standard Deviation.

Table S2. List of ASVs detected with mean relative abundances (%) over 0.1% in the group exposed to antibiotics.

ASV name	Exposed (mean Relative Abundance %)	Exposed (sd)
ASV3_Streptococcus	9.30	17.33
ASV2_Streptococcus	8.54	18.38
ASV1_Moraxella	6.54	13.29
ASV4_Haemophilus	4.18	9.76
ASV10_Haemophilus_influenzae	3.60	12.18
ASV18_Staphylococcus	3.11	8.80
ASV22_Streptococcus_pneumoniae	3.07	10.82
ASV11_Streptococcus	2.61	5.58
ASV7_Moraxella	2.36	4.76
ASV12_Streptococcus	2.32	4.51
ASV37_Staphylococcus	1.95	5.61
ASV87_Streptococcus	1.67	10.35
ASV6_Dolosigranulum_pigrum	1.42	4.13
ASV5_Moraxella_nonliquefaciens	1.36	4.77
ASV23_Corynebacterium	1.29	3.20
ASV16_Haemophilus_influenzae	1.26	4.03
ASV15_Chryseobacterium	1.22	8.98
ASV608_Moraxella	1.21	8.89
ASV14_Haemophilus	1.19	2.93
ASV30_Haemophilus	1.13	3.97
ASV43_Staphylococcus	0.97	2.75
ASV28_Delftia	0.96	6.70
ASV85_Streptococcus_peroris	0.95	5.47
ASV57_Veillonella_dispar	0.93	2.15
ASV9_Haemophilus	0.84	2.95

ASV27_Streptobacillus_hongkongensis	0.76	5.10
ASV183_Mycoplasma_pneumoniae	0.73	5.38
ASV46_Staphylococcus	0.66	3.17
ASV58_Pantoea	0.66	4.83
ASV5411_Moraxella	0.65	4.78
ASV94_Streptococcus_salivarius	0.62	2.12
ASV47_Prevotella_melaninogenica	0.60	1.44
ASV1610_Peptoniphilus_gorbachii	0.56	4.14
ASV35_Chryseobacterium_gleum	0.56	4.15
ASV5418_Staphylococcus	0.56	1.83
ASV8_Moraxella_lincolnii	0.49	2.07
ASV5410_Streptococcus	0.47	2.49
ASV78_Haemophilus_influenzae	0.47	1.86
ASV5971_Pseudomonas	0.42	1.53
ASV17_Dolosigranulum	0.40	1.36
ASV121_Veillonella	0.40	1.70
ASV5407_Moraxella	0.39	2.88
ASV64_Granulicatella	0.37	0.66
ASV13_Moraxella	0.36	1.12
ASV8638_Haemophilus	0.34	1.53
ASV522_Rothia_mucilaginosa	0.34	2.17
ASV109_Moraxella	0.33	2.45
ASV29_Haemophilus	0.33	1.23
ASV9299_Streptococcus	0.33	1.63
ASV86_Prevotella_nanceiensis	0.31	1.87
ASV40_Chryseobacterium	0.31	2.27
ASV50_Acinetobacter_junii	0.30	2.18
ASV2154_Haemophilus_influenzae	0.30	2.11
ASV5420_Moraxella	0.27	2.02
ASV5436_Porphyromonas	0.26	1.46
ASV75_Lautropia_mirabilis	0.26	1.52
ASV54_Dolosigranulum	0.26	1.84
ASV49_Lachnospiraceae	0.25	1.82
ASV62_Delftia	0.23	1.62
ASV95_Dolosigranulum	0.22	1.64
ASV53_Veillonella	0.22	0.95
ASV236_Snodgrassella	0.22	1.04
ASV124_Abiotrophia_defectiva	0.22	0.63
ASV554_Fusobacterium_necrophorum	0.22	1.58
ASV491_Mycoplasma	0.20	1.48
ASV60_Acinetobacter	0.20	1.47
ASV9298_Streptococcus	0.20	1.17
ASV80_Kingella	0.20	1.41
ASV38_Alloprevotella	0.19	0.71
ASV144_Pantoea	0.18	1.34
ASV88_Corynebacterium	0.18	0.40
ASV626_Corynebacterium	0.18	0.98
ASV26_Haemophilus	0.18	0.79
ASV9289_Corynebacterium	0.18	0.83
ASV697_Staphylococcus_hominis	0.16	0.83

ASV25_Haemophilus_influenzae	0.16	1.20
ASV5444_Peptoniphilus	0.16	1.20
ASV34_Haemophilus_influenzae	0.16	1.19
ASV475_Prevotella	0.16	1.14
ASV166_Enterobacteriaceae	0.16	1.15
ASV103_Streptophyta	0.15	0.89
ASV5428_Haemophilus	0.15	1.11
ASV265_Staphylococcus_epidermidis	0.15	0.69
ASV100_Porphyromonas	0.14	0.48
ASV5423_Haemophilus	0.14	1.03
ASV9295_Haemophilus	0.14	1.01
ASV31_Haemophilus_influenzae	0.13	0.68
ASV9312_Staphylococcus	0.13	0.97
ASV2294_Staphylococcus	0.13	0.71
ASV9297_Streptobacillus	0.13	0.96
ASV150_Oribacterium_sinus	0.13	0.83
ASV2126_Haemophilus_influenzae	0.13	0.95
ASV73_Acinetobacter	0.13	0.74
ASV98_Acidovorax	0.12	0.46
ASV111_Veillonella	0.12	0.79
ASV118_Haemophilus_parainfluenzae	0.12	0.30
ASV167_Porphyromonas	0.12	0.60
ASV374_Streptobacillus	0.12	0.81
ASV247_Staphylococcus	0.12	0.51
ASV1299_Sphingomonas	0.11	0.30
ASV9294_Haemophilus	0.11	0.82
ASV546_Mycoplasma	0.11	0.83
ASV102_Neisseria	0.11	0.39
ASV188_Staphylococcus_hominis	0.11	0.42
ASV257_Rothia_mucilaginosa	0.11	0.50
ASV114_Alloprevotella	0.11	0.79
ASV89_Alloprevotella	0.11	0.24
ASV130_Veillonella	0.10	0.26
ASV32_Haemophilus_influenzae	0.10	0.76
ASV728_Neisseria	0.10	0.73
ASV9305_Veillonella	0.10	0.43
ASV5409_Moraxella	0.10	0.74

Abbreviations: ASV; Amplicon Sequence Variant. SD; Standard Deviation.

Figure S1. Phylogenetic tree of streptococcal type-strains. Phylogenetic tree showing the positions of main streptococcal ASVs detected in our study (ASV2, ASV3, ASV11 and ASV12) relative to other type-strains within the genus *Streptococcus*. The tree was reconstructed by the maximum-likelihood method based on V3-V4 region of 16S rRNA gene sequences. The cell collection precedence and their corresponding GenBank accession numbers for 16S rRNA genes are included for each type-strain.

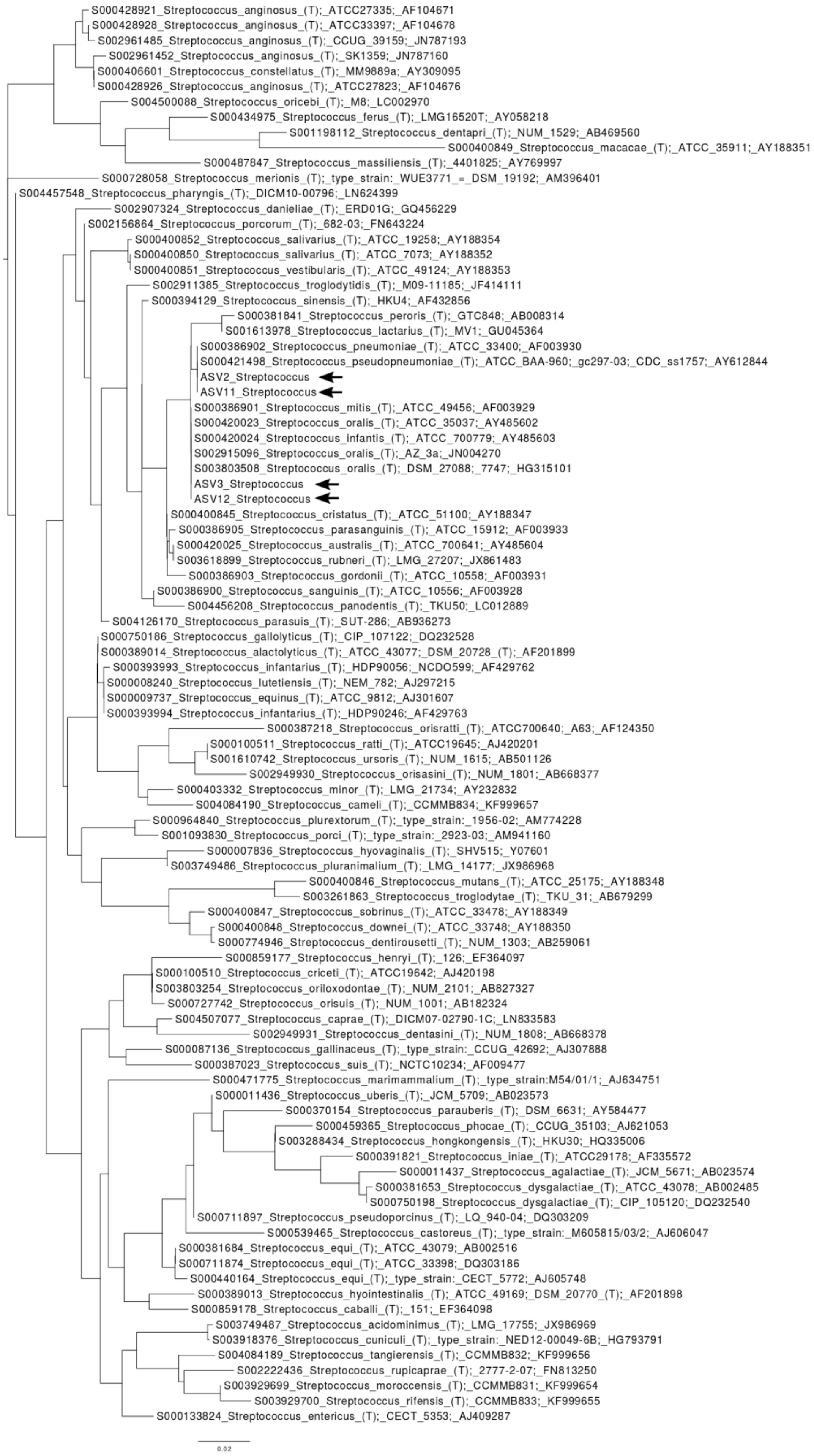
S000428921_Streptococcus_anginosus_(T);_ATCC27335;_AF104671
S000428928_Streptococcus_anginosus_(T);_ATCC33397;_AF104678
S002961485_Streptococcus_anginosus_(T);_CCUG_39159;_JN787193
S002961452_Streptococcus_anginosus_(T);_SK1359;_JN787160
S000406601_Streptococcus_constellatus_(T);_MM9889a;_AY309095
S000428926_Streptococcus_anginosus_(T);_ATCC27823;_AF104676
S004500088_Streptococcus_oricebi_(T);_M8;_LC002970
S000434975_Streptococcus_ferus_(T);_LMG16520T;_AY058218
S001198112_Streptococcus_dentapri_(T);_NUM_1529;_AB469560
S000400849_Streptococcus_macacae_(T);_ATCC_35911;_AY188351
S000487847_Streptococcus_massiliensis_(T);_4401825;_AY769997
S000728058_Streptococcus_merionis_(T);_type_strain:_WUE3771_=_DSM_19192;_AM396401
S004457548_Streptococcus_pharyngis_(T);_DICM10-00796;_LN624399
S002907324_Streptococcus_danieliae_(T);_ERD01G;_GQ456229
S002156864_Streptococcus_porcorum_(T);_682-03;_FN643224
S000400852_Streptococcus_salivarius_(T);_ATCC_19258;_AY188354
S000400850_Streptococcus_salivarius_(T);_ATCC_7073;_AY188352
S000400851_Streptococcus_vestibularis_(T);_ATCC_49124;_AY188353
S002911385_Streptococcus_troglodytidis_(T);_M09-11185;_JF414111
S000394129_Streptococcus_sinensis_(T);_HKU4;_AF432856
S000381841_Streptococcus_peroris_(T);_GTC848;_AB008314
S001613978_Streptococcus_lactarius_(T);_MV1;_GU045364
S000386902_Streptococcus_pneumoniae_(T);_ATCC_33400;_AF003930
S000421498_Streptococcus_pseudopneumoniae_(T);_ATCC_BAA-960;_gc297-03;_CDC_ss1757;_AY612844
ASV2_Streptococcus
ASV11_Streptococcus
S000386901_Streptococcus_mitis_(T);_ATCC_49456;_AF003929
S000420023_Streptococcus_oralis_(T);_ATCC_35037;_AY485602
S000420024_Streptococcus_infantis_(T);_ATCC_700779;_AY485603
S002915096_Streptococcus_oralis_(T);_AZ_3a;_JN004270
S003803508_Streptococcus_oralis_(T);_DSM_27088;_7747;_HG315101
ASV3_Streptococcus
ASV12_Streptococcus
S000400845_Streptococcus_cristatus_(T);_ATCC_51100;_AY188347
S000386905_Streptococcus_parasanguinis_(T);_ATCC_15912;_AF003933
S000420025_Streptococcus_australis_(T);_ATCC_700641;_AY485604
S003618899_Streptococcus_rubneri_(T);_LMG_27207;_JX861483
S000386903_Streptococcus_gordonii_(T);_ATCC_10558;_AF003931
S000386900_Streptococcus_sanguinis_(T);_ATCC_10556;_AF003928
S004456208_Streptococcus_panodentis_(T);_TKU50;_LC012889
S004126170_Streptococcus_parasuis_(T);_SUT-286;_AB936273
S000750186_Streptococcus_gallolyticus_(T);_CIP_107122;_DQ232528
S000389014_Streptococcus_alactolyticus_(T);_ATCC_43077;_DSM_20728_(T);_AF201899
S000393993_Streptococcus_infantarius_(T);_HDP90056;_NCDO599;_AF429762
S000008240_Streptococcus_lutetiensis_(T);_NEM_782;_AJ297215
S000009737_Streptococcus_equinus_(T);_ATCC_9812;_AJ301607
S000393994_Streptococcus_infantarius_(T);_HDP90246;_AF429763
S000387218_Streptococcus_orisratti_(T);_ATCC700640;_A63;_AF124350
S000100511_Streptococcus_ratti_(T);_ATCC19645;_AJ420201
S001610742_Streptococcus_ursoris_(T);_NUM_1615;_AB501126
S002949930_Streptococcus_orisasini_(T);_NUM_1801;_AB668377
S000403332_Streptococcus_minor_(T);_LMG_21734;_AY232832
S004084190_Streptococcus_cameli_(T);_CCMMB834;_KF999657
S000964840_Streptococcus_plurextorum_(T);_type_strain:_1956-02;_AM774228
S001093830_Streptococcus_porci_(T);_type_strain:_2923-03;_AM941160
S000007836_Streptococcus_hyovaginalis_(T);_SHV515;_Y07601
S003749486_Streptococcus_pluranimalium_(T);_LMG_14177;_JX986968
S000400846_Streptococcus_mutans_(T);_ATCC_25175;_AY188348
S003261863_Streptococcus_troglodytae_(T);_TKU_31;_AB679299
S000400847_Streptococcus_sobrinus_(T);_ATCC_33478;_AY188349
S000400848_Streptococcus_downei_(T);_ATCC_33748;_AY188350
S000774946_Streptococcus_dentirousetti_(T);_NUM_1303;_AB259061
S000859177_Streptococcus_henryi_(T);_126;_EF364097
S000100510_Streptococcus_criceti_(T);_ATCC19642;_AJ420198
S003803254_Streptococcus_oriloxodontae_(T);_NUM_2101;_AB827327
S000727742_Streptococcus_orisuis_(T);_NUM_1001;_AB182324
S004507077_Streptococcus_caprae_(T);_DICM07-02790-1C;_LN833583
S002949931_Streptococcus_dentasini_(T);_NUM_1808;_AB668378
S000087136_Streptococcus_gallinaceus_(T);_type_strain:_CCUG_42692;_AJ307888
S000387023_Streptococcus_suis_(T);_NCTC10234;_AF009477
S000471775_Streptococcus_marimammalium_(T);_type_strain:M54/01/1;_AJ634751
S000011436_Streptococcus_uberis_(T);_JCM_5709;_AB023573
S000370154_Streptococcus_parauberis_(T);_DSM_6631;_AY584477
S000459365_Streptococcus_phocae_(T);_CCUG_35103;_AJ621053
S003288434_Streptococcus_hongkongensis_(T);_HKU30;_HQ335006
S000391821_Streptococcus_iniae_(T);_ATCC29178;_AF335572
S000011437_Streptococcus_agalactiae_(T);_JCM_5671;_AB023574
S000381653_Streptococcus_dysgalactiae_(T);_ATCC_43078;_AB002485
S000750198_Streptococcus_dysgalactiae_(T);_CIP_105120;_DQ232540
S000711897_Streptococcus_pseudoporcinus_(T);_LQ_940-04;_DQ303209
S000539465_Streptococcus_castoreus_(T);_type_strain:_M605815/03/2;_AJ606047
S000381684_Streptococcus_equi_(T);_ATCC_43079;_AB002516
S000711874_Streptococcus_equi_(T);_ATCC_33398;_DQ303186
S000440164_Streptococcus_equi_(T);_type_strain:_CECT_5772;_AJ605748
S000389013_Streptococcus_hyointestinalis_(T);_ATCC_49169;_DSM_20770_(T);_AF201898
S000859178_Streptococcus_caballi_(T);_151;_EF364098
S003749487_Streptococcus_acidominimus_(T);_LMG_17755;_JX986969
S003918376_Streptococcus_cuniculi_(T);_type_strain:_NED12-00049-6B;_HG793791
S004084189_Streptococcus_tangierensis_(T);_CCMMB832;_KF999656
S002222436_Streptococcus_rupicaprae_(T);_2777-2-07;_FN813250
S003929699_Streptococcus_moroccensis_(T);_CCMMB831;_KF999654
S003929700_Streptococcus_rifensis_(T);_CCMMB833;_KF999655
S000133824_Streptococcus_entericus_(T);_CECT_5353;_AJ409287
0.02

Figure S2. Classification of children with IPD according to antibiotic-exposure using a Random Forest model based on nasopharyngeal microbiota composition at the genus level. The 16S rRNA data was merged at the genus level and all 344 genera detected were included as explanatory variables in the model. Confounding variables including age, gender, seasonality, vaccination, ICU admission, and length of hospital stay were also included in the model as copredictors. **A)** ROC curve showing the performance of the RF model for patient's classification using relative abundances of bacterial genera and confounding variables. **B)** Barplot showing top 25 most important features to class separation according to Mean Decrease Accuracy score (confounding variables were not found among the top important features). Color-coding shows directionality of the association for each of the 25 top features to either the not-exposed (yellow) or ATB-exposed (blue) group based on a post-hoc analyses with cliff's delta estimation of the effect size. In addition, a heatmap displaying relative abundance (%) of these top 25 features across samples is shown at the right. Each column represents a sample, while each row represents a different feature. In the x axis, samples are split by group and ordered according to hierarchical clustering using a Bray-Curtis dissimilarity measure.

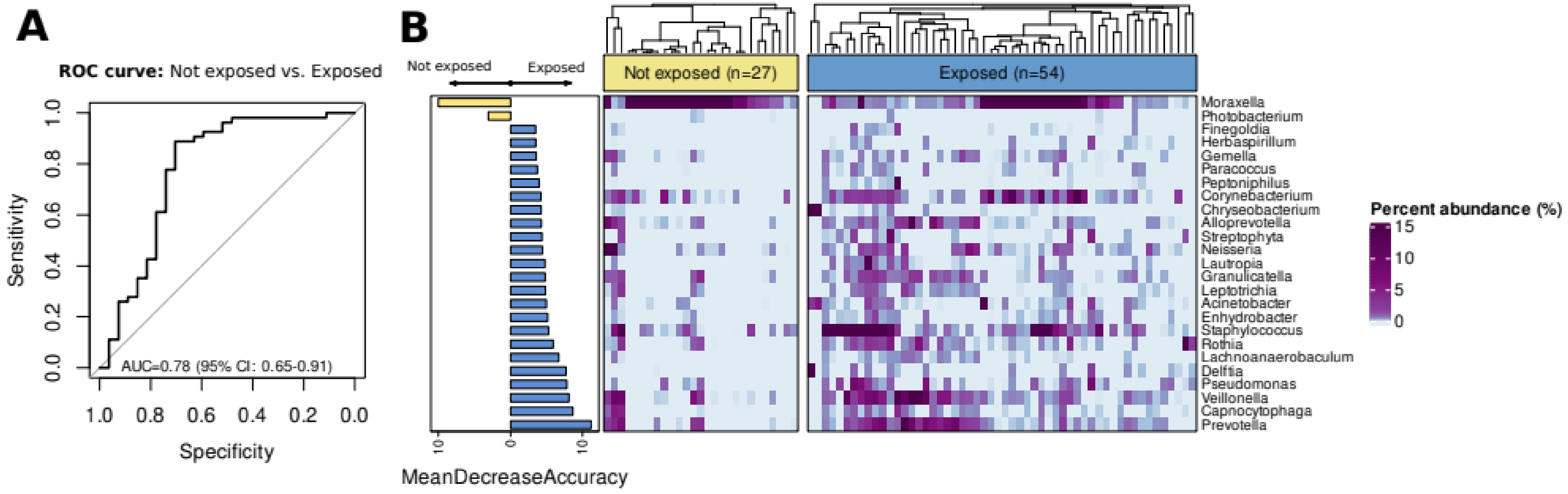

Figure S3. Differential ranking analysis with Songbird. Samples from the group of patients with IPD exposed to antibiotics were utilized as the reference to infer differentially abundant ASVs according to antibiotic exposure. The ASVs with the highest differentials (differentials >1) are colored according to the direction of the association; features colored in blue are more abundant in samples from patients exposed to antibiotics, while features colored in yellow are more abundant in samples from patients not exposed to antibiotics. Differential ranking analysis was performed with the Songbird standalone tool (https://github.com/biocore/songbird) using default parameters and results were plotted with ggplot2.

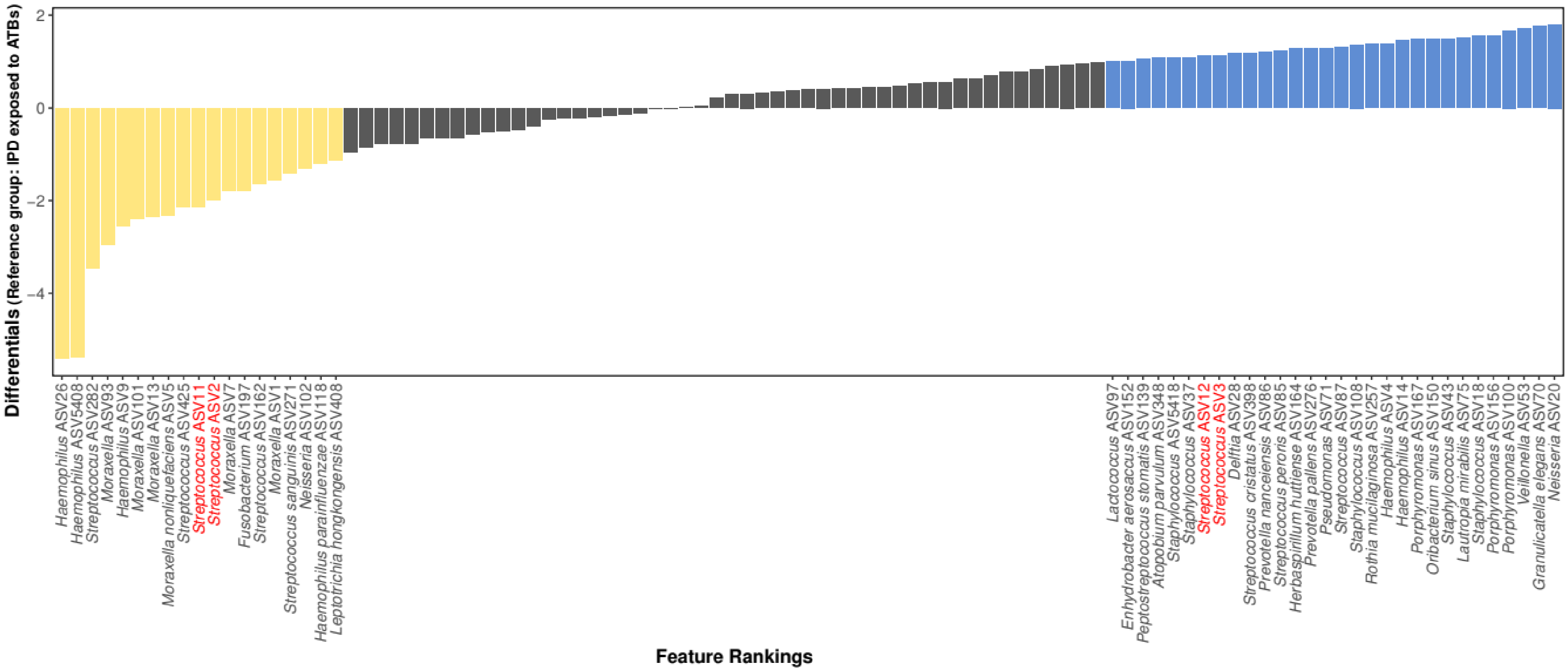

Supplement: Supplementary file 1 [file DataSheet_1.pdf]
